# Reply 1: Call for NICE to review urgently their guidance concerning first-line chemotherapy for metastatic colorectal cancer

**DOI:** 10.1038/sj.bjc.6600849

**Published:** 2003-04-01

**Authors:** M P Saunders, J W Valle

**Affiliations:** 1 Department of Clinical Oncology, Christie Hospital NHS Trust, Wilmslow Road, Withington, Manchester, M20 9BX, UK

**Sir**,

In our editorial ‘Why hasn't the National Institute been NICE to patients with colorectal cancer’, we highlighted the disparity of treatment in England and Wales compared to other industrialised countries, particularly with respect to the poor 5-year survival figures. Our concerns focused on the conclusions reached by the NICE panel in the face of two prospective, randomised trials involving more than 1000 patients showing a survival advantage in favour of first-line irinotecan-based therapy (Douillard *et al*, 2000; Saltz *et al*, 2000).

We only referred to the MRC CRO8 (FOCUS) study since the NICE panel recommended entering patients into this trial and were planning to review their guidance when the results from this study are available. We never questioned the ethical validity of the FOCUS study. We simply pointed out that NICE seem to have linked their guidance very closely to this study and feel that they have frankly misinterpreted the many positive studies from here and abroad. NICE also failed to appreciate that patients who were randomised into the two oxaliplatin arms were unable to receive irinotecan at any point, even though they supported second-line use of this drug. We were not questioning the FOCUS study; we simply alluded to this disparity created by NICE. Since our editorial was published, the FOCUS study has, in our opinion, quite rightly added a third-tier of treatment, so allowing all patients to receive both oxaliplatin and irinotecan at some point. However, according to NICE recommendations, patients who prefer not to be entered into this study should only receive 5-Fluorouracil and folinic acid (5FU/FA) ‘up-front’, unless they fall into the very small group of patients that have potentially operable liver metastases.

With the widespread availability of the internet and because we need to provide enough information to allow patients to give informed consent, they are quite rightly questioning the NICE guidance in a similar manner to the 28 colorectal oncologists who wrote to the Daily Telegraph in June. Patients are also rightly concerned that the NICE guidance was influenced too much by government funding constraints rather than clinical-effectiveness. We would strongly urge NICE to consider a *fresh review* before 2005, based on both the convincing existing data (Douillard *et al*, 2000; Saltz *et al*, 2000) and the rapidly emerging new data using all three drugs planned or unplanned (Tournigand *et al*, 2001; Goldberg *et al*, 2002).
